# Towards clinical implementation of circulating tumor DNA in metastatic prostate cancer: Opportunities for integration and pitfalls to interpretation

**DOI:** 10.3389/fonc.2022.1054497

**Published:** 2022-11-10

**Authors:** Edmond M. Kwan, Alexander W. Wyatt, Kim N. Chi

**Affiliations:** ^1^ Vancouver Prostate Centre, Department of Urologic Sciences, The University of British Columbia, Vancouver, BC, Canada; ^2^ BC Cancer, Vancouver Centre, Vancouver, BC, Canada; ^3^ Michael Smith Genome Sciences Centre, BC Cancer, Vancouver, BC, Canada; ^4^ Department of Medicine, The University of British Columbia, Vancouver, BC, Canada

**Keywords:** cell-free DNA, CtDNA, biomarker, resistance, targeted therapy

## Abstract

Plasma circulating tumor DNA (ctDNA) represents short fragments of tumor-derived DNA released into the bloodstream primarily from cancer cells undergoing apoptosis. In metastatic castration-resistant prostate cancer (mCRPC), characterizing genomic alterations in ctDNA identifies mutations, copy number alterations, and structural rearrangements with predictive and prognostic biomarker utility. These associations with clinical outcomes have resulted in ctDNA increasingly incorporated into routine clinical care. In this review, we summarize current and emerging applications for ctDNA analysis in metastatic prostate cancer, including outcome prediction, treatment selection, and characterization of treatment resistance. We also discuss potential pitfalls with interpreting ctDNA findings, namely false negatives arising from low tumor content and optimal assay design, including correction for clonal hematopoiesis of indeterminate potential and germline variants. Understanding the influence of these limitations on interpretation of ctDNA results is necessary to overcome barriers to clinical implementation. Nevertheless, as assay availability and technology continue to improve, recognizing both opportunities and shortcomings of ctDNA analysis will retain relevance with informing the implementation of precision-oncology initiatives for metastatic prostate cancer.

## Introduction

Blood-based liquid biopsies are a minimally invasive tool to acquire molecular insights in metastatic prostate cancer. Diverse tumor-derived products can be detected in metastatic castration-resistant prostate cancer (mCRPC), including circulating tumor cells, circulating tumor DNA/RNA, proteins, extracellular vesicles, and tumor-educated platelets (1). Of these, plasma circulating tumor DNA (ctDNA) has attracted the greatest attention. Ease of sampling (including serial collection) and well-established isolation protocols have fueled considerable efforts to understand its value as a prognostic, predictive and response biomarker in the advanced disease setting. With the recent US Food and Drug Administration (FDA) approval of multiple commercial assays with companion diagnostic status, ctDNA is increasingly being used for the genomic profiling of patients as part of standard-of-care practice.

Plasma cell-free DNA (cfDNA) is highly fragmented, double-stranded extracellular DNA released into the bloodstream primarily from cells undergoing apoptosis (2). In healthy individuals, most cfDNA comes from normal turnover of white blood cells (3), with concentrations ranging from 1-10ng per milliliter of blood (4). In contrast, cfDNA concentration in patients with mCRPC is greater by two-to-three-fold (5). The abundance of ctDNA (*i.e.*, tumor-derived cfDNA) as a proportion of total cell-free DNA can be expressed as the ctDNA fraction (ctDNA%) of a blood sample. The short half-life of ctDNA (minutes to hours) further lends itself as a real-time assessment of the current disease state.

With a growing number of therapy options now available to patients with metastatic prostate cancer, tools that guide treatment selection and sequencing remain an unmet clinical need. In this review, we summarize current and emerging ctDNA applications for outcome prediction, treatment selection and disease monitoring in metastatic prostate cancer. We focus on correlative studies that provide a framework for integrating ctDNA profiling into routine patient care. Importantly, we highlight potential pitfalls with interpreting ctDNA findings in challenging scenarios. As assay availability and technology improve, recognizing both opportunities and shortcomings of ctDNA analysis will become increasingly relevant to supporting precision-based care of patients with metastatic prostate cancer.

### Approaches to genotyping in metastatic prostate cancer

Genomic biomarker testing in metastatic prostate cancer normally requires analysis of tumor tissue from prostate primary specimens (6). However, disease-specific factors complicate using tissue as source material. In a recent analysis of over 4000 prostate cancer tissue specimens, sequencing failure rates approached 30-40% (7). Low pathological tumor content and poor DNA yield were frequently responsible for unsuccessful sequencing, an issue that pervasively impacts prostate core needle biopsies obtained at initial diagnosis. Furthermore, marked genomic spatial heterogeneity within the prostate can undermine the ability of a single prostate biopsy specimen to correctly identify the dominant genotype responsible for metastatic disease development (8, 9). In patients with metachronous metastatic disease, substantial time can separate initial diagnosis and subsequent development of clinical metastases (10). During this period, tumor evolution shaped by systemic therapy exposure to androgen deprivation therapy (ADT), docetaxel chemotherapy and/or potent androgen receptor pathway inhibitors (11) (ARPI) mean that archival tissue may not reflect the contemporaneous tumor genotype. One striking example involves genetic alterations in the androgen receptor (*AR*). *AR* alterations are rare in systemic therapy-naive prostate cancer, but highly enriched in the castration-resistant disease setting (5, 12–16). Furthermore, *AR* can exhibit heterogeneity between metastases within the same patient (17, 18).

Overcoming these limitations of primary tissue analysis by obtaining fresh biopsies from metastatic disease sites is similarly problematic. Not only are metastatic biopsies technically challenging, but serial sampling is impractical for patients and health services alike. Critically, single lesion sampling fails to represent the diversity of competing tumor clones present between different disease sites. In a recent study employing deep whole-genome sequencing of serial plasma samples and tissue biopsies of synchronous metastases in poor-risk mCRPC patients, the contribution of any singular metastasis to the total ctDNA pool was low, providing evidence to support the long held notion that ctDNA captures tumor material from multiple metastatic foci (19). This ability to capture spatial and temporal heterogeneity between metastases have led to a number of clinical applications for ctDNA in metastatic prostate cancer.

## Clinical applications

Considerable infrastructural, technological, and intellectual overhead generally accompanies most ctDNA analysis platforms. These resourcing requirements mean that a majority of clinicians around the world continue to have limited access to ctDNA profiling to inform patient care. Instead, clinicians typically encounter ctDNA testing in the clinical trial setting, where it is increasingly used alongside tumor tissue testing to screen for genomic alterations that may confer sensitivity to established and novel targeted therapies (20–25). Outside of clinical trials, US FDA-approved commercial platforms are available (e.g., FoundationOne Liquid CDx, Guardant360 CDx) (26, 27), but can be cost prohibitive for patients unable to acquire partial or full financial reimbursement for testing. While some academic research centers offer in-house ctDNA assays (28), these services are beyond reach for most community oncologists. Finally, where testing is available, there remains no consensus surrounding best practices for the post-analytic phase of ctDNA profiling. In contrast to considerable efforts to harmonize pre-analytical variables associated with cfDNA/ctDNA collection and isolation (29), the optimal approach to evaluation, interpretation, reporting and integration of patient results into clinical care is not established (30). Collectively, these factors must be addressed before widespread adoption of ctDNA testing to inform routine patient care. Despite these challenges, several clinical applications are likely to emerge as forerunners for ctDNA implementation in management of advanced prostate cancer.

### ctDNA for prognostication

Accurate prognostic information informs systemic therapy selection, dictates treatment urgency, stratifies participants in clinical trials, and shapes discussions with patients around expected long-term outcomes (31, 32). Current survival prediction models incorporate pathologic, biochemical and radiographic markers of disease burden, including performance status, Gleason score, prostate specific antigen (PSA) levels, lactate dehydrogenase, albumin, alkaline phosphatase, number of bone metastases, and visceral organ involvement (33–36). While these models are informative, performance is variable, and most were validated in an era with few life-prolonging treatment options. New methods of prognostication are therefore needed to account for a rapidly shifting therapeutic landscape.

#### Prognostic value of ctDNA%

In mCRPC, both total cfDNA concentration and ctDNA% correlate with many conventional clinical markers of disease burden, including visceral disease, lactate dehydrogenase, hemoglobin and alkaline phosphatase levels (5, 16, 37–39). However, even when accounting for these poor-risk clinical features, higher pretreatment cfDNA concentration and ctDNA% independently predicts shorter progression-free and overall survival in patients commencing contemporaneous systemic therapies. These associations are evident in men receiving next-generation ARPIs such as abiraterone and enzalutamide (5, 40–45), as well as taxane cytotoxic therapies docetaxel and cabazitaxel (16, 39, 46, 47). Less well understood is the precise relationship between these metrics and life expectancy. Despite knowledge of their negative prognostic potential, no clinically relevant thresholds for cfDNA concentration and ctDNA% currently exist. In addition, the influence of prior systemic treatment on the interpretation of ctDNA% has not been systematically investigated. This is particularly relevant in metastatic castration-sensitive prostate cancer (mCSPC), where ADT administration results in rapid ctDNA% decline (48). Understanding this, along with the additive contribution of pretreatment cfDNA concentration and ctDNA% to existing clinical prognostic models is needed before integration in the clinical setting.

#### Quantification of ctDNA

**Figure 1:**
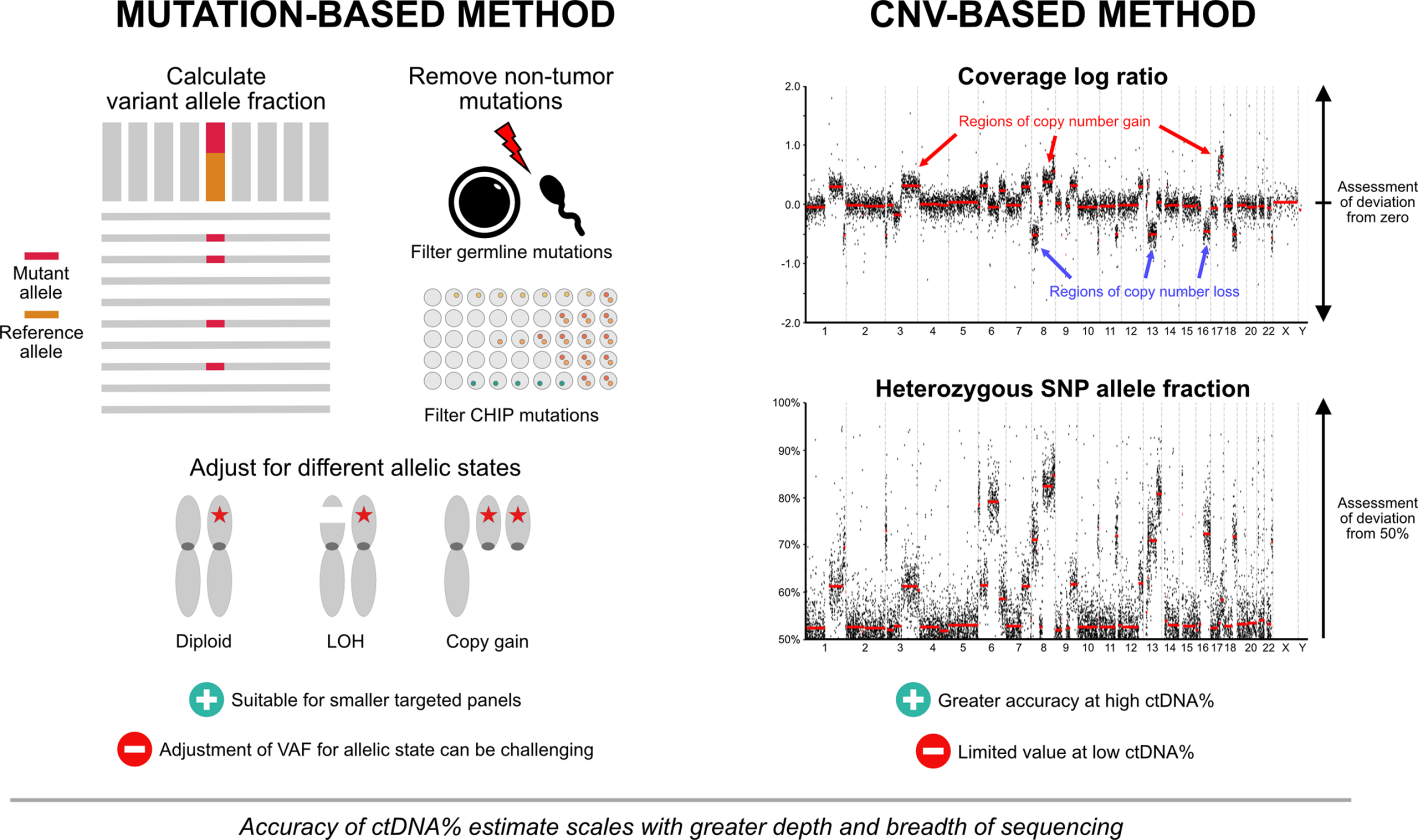
Approaches to ctDNA fraction estimation. Both mutation-based and copy number-based approaches are used for ctDNA% estimation. Mutation-based approaches typically utilize the variant allele fraction of truncal mutation(s), with or without adjustment for different allelic configurations. Germline mutations and mutations arising from clonal hematopoiesis of indeterminate potential are removed to prevent contribution to ctDNA% estimates. Mutation-based methods can be complemented by systematic assessments of genomic regions affected by copy number changes. Regions of copy gain or loss are identified by evaluating sequencing read depth ratios and/or shifts in B-allele frequency at germline heterozygous SNP loci. The degree of signal from segment-level copy number alterations can then be used with overall tumor ploidy estimates to calculate ctDNA%. Panels that provide both mutation and copy number data encompassing broad genomic territory are likely to yield the most accurate estimates across a spectrum of ctDNA%. Abbreviations: CHIP, clonal hematopoiesis of indeterminate potential; ctDNA%, circulating tumor DNA fraction; LOH, loss-of-heterozygosity; SNP, single nucleotide polymorphism; VAF, variant allele fraction.

A challenge with incorporating ctDNA% as a routine biomarker is that various methods exist for quantifying plasma ctDNA% (**Figure 1**). At its simplest form, ctDNA% can be estimated from the variant allele frequency (VAF) of somatic mutations detected within a patient sample. Typically, the highest VAF mutation(s) are chosen for ctDNA% approximation, on the assumption that they likely represent truncal mutation(s) present in most ctDNA-releasing cancer cells. Advances in molecular barcoding of individual cfDNA fragments, and custom-designed patient-specific custom panels (using mutations found in primary tissue) coupled with ultra-deep targeted sequencing have enabled mutations to be detected well below 1% VAF, allowing more refined estimates of ctDNA% (49). Mutation-based ctDNA% estimation methods can be complemented by systematic assessments of genomic regions affected by copy number variants (CNV). Current computational algorithms identify regions of copy gain or loss by evaluating sequencing read depth ratios (available from both targeted and shallow whole-genome sequencing approaches) and/or shifts in B-allele frequency at germline heterozygous SNP loci (requiring targeted sequencing across specific SNPs). The degree of signal from segment-level copy number alterations can then be used with overall tumor ploidy estimates to calculate ctDNA%, similar to how magnitude of somatic mutation VAF corresponds to ctDNA% (50, 51).

No single method of ctDNA quantification is appropriate for all samples. Ideally, panels that provide both mutation and CNV data will yield the most accurate estimates across a spectrum of ctDNA%. Mutation-based methods are preferable in lower tumor content samples, as CNVs are difficult to differentiate from background signal at <10-15% ctDNA. Conversely, CNV-based (or adjusted) methods may be preferred in instances where use of mutation VAF alone will likely lead to ctDNA% overestimates, such as tumor aneuploidy (*e.g.*, whole genome duplication), loss-of-heterozygosity across the wild-type allele, and copy gain of the allele carrying the mutation (known as mutant allele specific imbalance) (52). False positive mutations affecting ctDNA% estimates can also arise in patients with clonal hematopoiesis of indeterminate potential (CHIP). CHIP is defined by the presence of clonal expansion of somatic mutations in hematopoietic stem cells in the absence of any concurrent hematologic neoplasia. The risk of misattributing a CHIP-related somatic mutation as a legitimate prostate cancer mutation may be as high as 15-20% (53), and can lead to an overestimate of ctDNA%. Concurrent sequencing of matched white blood cells is a simple method to ensure that CHIP-related mutations are correctly identified, but surprisingly is not performed by current commercially available clinical grade ctDNA tests. Importantly, confidence in ctDNA% estimates scale with sequencing depth and breath. Sequencing approaches that encompass broad genomic territory (*e.g.*, larger pan-cancer targeted panels or whole exome/genome assays) produce a greater amount of data that can be used for estimates. In turn, this must be balanced with the financial toxicity and substantial human capital required for successful implementation across a wide population group. While the real-world cost of shallow whole genome sequencing (0.1-5x depth) continues to fall with time, higher sequencing depth (>20-40x depth) is likely required to provide sufficient biologic insights that inform treatment decision-making (19, 54). Factoring in the expenditure associated with data analysis, interpretation and patient counseling, the sum cost of informative whole genome analysis is likely to exceed $10,000-$20,000 per patient (55), arguably not cost-effective for routine implementation in all advanced prostate cancer patients.

### ctDNA for response prediction

Serial PSA measurement is a mainstay of disease monitoring in metastatic prostate cancer. In most patients with mCSPC, PSA falls rapidly after starting ADT, with nadir levels prognostic for overall survival (56). In mCRPC, early PSA decline at four weeks following ARPI commencement predicts durability of treatment response and overall survival, irrespective of prior taxane chemotherapy (57). Nevertheless, PSA is an imperfect response marker. Up to 25% of patients receiving ADT +/- docetaxel for mCSPC experience clinical progression without PSA elevation (58). Similar findings have also been described in enzalutamide-treated mCRPC (59). Importantly, heavily pretreated mCRPC enriches AR-independent disease phenotypes well-recognized to secrete low levels of PSA (60).

Radiographic disease monitoring also has limitations. The sensitivity and specificity of CT and bone scan imaging is suboptimal for accurate identification of lymph node and skeletal metastases alike (61). The phenomenon of bone flare whereby effective treatments lead to the apparent appearance of new or more prominent bone lesions can be misinterpreted as true progression. While new radionuclide imaging techniques such as PSMA PET/CT may address the limitations of conventional imaging, high level evidence supporting prospective use as a treatment evaluation tool is lacking (62).

#### Monitoring ctDNA% for response prediction

Tracking ctDNA% has the potential to address shortcomings of PSA and radiographic disease assessment. Notably, while on-treatment PSA and ctDNA% generally trend in the same direction, there is weak correlation for the magnitude of this change (38, 63). This suggests that PSA and ctDNA% do not reflect the same tumor biology, and ctDNA% could provide additional information to support current clinical assessment markers.

In patients receiving systemic therapy for mCRPC, early reductions in cfDNA concentration or ctDNA% (within 4-8 weeks of treatment commencement) are independently associated with longer progression-free survival and overall survival (47, 63–65). Conversely, a rise in ctDNA% at 12 weeks significantly increases the risk of early biochemical and radiographic progression on ARPI or cabazitaxel chemotherapy (16, 66). Examples where ctDNA% correctly differentiates between radiographic flare phenomenon and true progression have also been reported in patients receiving ARPI or immune checkpoint inhibitor therapy (66, 67).

The depth of ctDNA% decline may also be relevant. In an analysis of patients receiving abiraterone +/- ipatasertib, deeper reductions in ctDNA% were associated with higher rates of partial or complete radiographic soft tissue responses to therapy (64). Relatedly, two recent reports suggest that clearance of ctDNA from circulation may be the most informative predictor of long-term clinical outcomes. Tracking alterations in prostate cancer driver genes *PTEN*, *TP53* and *RB1*, Jayaram et al. showed that persistent detection after four weeks of ARPI was independently associated with worse survival (68). Similarly, Tolmeijer et al. found that mCRPC patients with detectable ctDNA at both baseline and four-week timepoints following ARPI commencement selected individuals more likely to experience rapid acquired resistance within six months of starting treatment (65). Both studies found that patients that convert from ctDNA detectable to undetectable had outcomes similar to patients that were ctDNA undetectable at both timepoints. Note that ‘detectable’ versus ‘undetectable’ is a blood volume-dependent and assay-dependent variable. Greater volume of blood collected, and higher sequencing depth increases the probability of detecting rare tumor-derived cfDNA fragments. In future, more sensitive methodological approaches including personalized tumor-informed mutation panels and methylation-based cfDNA profiling are likely further reduce the absolute lower limit of detection for ctDNA (49), although tests that classify all patients as ctDNA-positive are unlikely to be useful in guiding patient management. Further work is required to understand exact clinically meaningful thresholds for ctDNA% detection or declines. In addition, we need to better ascertain the ideal on-treatment timepoint(s) to monitor ctDNA%. Finally, understanding how interpretation of ctDNA changes differs according to disease state (*e.g.*, mCSPC *vs* mCRPC) and class of systemic therapy will require further evaluation as exploratory intermediate endpoints in large prospective cohorts.

### ctDNA for guiding precision oncology therapies

The genomic landscape of metastatic prostate cancer has been established over a series of coordinated tissue sequencing efforts (12–15, 69, 70). These studies identified potentially actionable alterations in key growth pathways targetable using precision medicines. To date, two classes of targeted therapies have gained regulatory approval in the biomarker-selected mCRPC population: poly(ADP-ribose) polymerase (PARP) inhibitors olaparib and rucaparib in tumors with defective homologous recombination repair (HRR), and the anti-PD1 immune checkpoint inhibitor pembrolizumab in tumors with deficient mismatch repair (MMR) and/or high mutational burden.

The convenience of collecting ctDNA has spurred hopes that it could eventually replace tissue as a means for screening some patients for targeted therapies. The full spectrum of genomic alterations can be detected in ctDNA, including mutations, copy number alterations and structural rearrangements. However, like tumour tissue, liquid biopsy similarly suffers from sample insufficiency due to low levels of tumor material in the blood. Depending on prior treatment exposure and burden of disease, it is estimated that ctDNA fraction is below 1% in between 30-40% of patients with mCRPC (5, 41, 42); these patients are not amenable to tumor genotyping using ctDNA. In general, providing sufficient ctDNA% is present, ctDNA alterations from patients with mCRPC show strong concordance with matched metastatic tissue (19, 71), and reproduce the expected mutational landscape (5, 16, 42, 43, 71–73). Apparent differences are explained by low ctDNA% samples, variability in assay design and presence of subclonal alterations. The impact of these factors on identifying metastatic prostate cancer patients suitable for PARP inhibitors and immunotherapy is discussed below.

#### Homologous recombination repair deficiency

PARP inhibitors are standard-of-care in prostate cancer harboring pathogenic alterations in HRR-related genes (74–76). Platinum chemotherapy has also been shown to be effective in this genomic subgroup (77). 15-20% of mCRPC harbor deleterious HRR-related gene mutations, most commonly in *BRCA2*, *ATM* and *CDK12*. Concordance between tissue and ctDNA for HRR gene mutations is excellent, exceeding 90% in most studies (23, 64, 71, 78, 79). However, an HRR-related gene mutation alone may be insufficient to confer therapeutic vulnerability. Biallelic inactivation is increasingly recognized as a critical prerequisite of functional HRR deficiency, and by virtue, promoting greatest susceptibility to PARP inhibitors (76, 80). The genomic insult that establishes biallelic inactivation is gene-dependent. For example, the mechanism of biallelic *BRCA2* inactivation in prostate cancer is primarily loss-of-heterozygosity (*i.e.*, copy deletion of the wildtype allele), but with homozygous deletion a clear secondary mechanism (81). Conversely, biallelic *CDK12* inactivation manifests almost exclusively as multiple separate somatic mutational events (78). As sensitivity for all major genomic alteration types in ctDNA is strongly influenced by tumor purity, confidence in ctDNA test results reporting HRR gene alterations must account for ctDNA%. Many commercial and academic plasma cfDNA assays are capable of detecting somatic mutations below 1% ctDNA, but heterozygous and homozygous deletions typically require far greater ctDNA to be reliably detected (78, 82, 83). Therefore, low ctDNA% may be a limiting factor in detecting HRR alterations in cfDNA, especially large structural rearrangements and homozygous deletion events (23).

Separate from somatic alterations, up to half of HRR-related gene alterations are germline in origin (84). Concurrent matched white blood cell DNA sequencing at the time of cfDNA profiling can readily discriminate between patients with germline versus somatic HRR gene mutations, with implications not just for PARP inhibitor candidacy, but also hereditary cancer screening. Unfortunately, many commercial ctDNA tests do not routinely perform concurrent germline screening. This failure also means that some CHIP-related mutations falling in HRR genes are at risk of being attributed to cancer and could influence PARP inhibitor eligibility. In a recent study of 69 men with advanced prostate cancer undergoing paired plasma cfDNA whole-blood germline control sample analysis, 10% had CHIP variants in genes used for US FDA-approved PARP inhibitors, most frequently in ATM (53). Without concurrent germline screening, these patients could inappropriately receive therapies including olaparib, rucaparib and niraparib, but would otherwise not be expected to respond to treatment.

#### Mismatch repair deficiency and somatic hypermutation

MMR genes (*MSH2*, *MSH6*, *MLH1* and *PMS2*) are responsible for encoding proteins that reverse base substitution errors arising from DNA replication. MMR-deficient prostate tumors are associated with unstable genomes, manifesting as somatic hypermutation and microsatellite instability (85). This increase in somatic mutations generates tumor neoantigens, which may improve immune system recognition and provides rationale for use of immune checkpoint inhibitors. Evidence to this, durable responses to PD-1 inhibitors have been reported in MMR-deficient mCRPC (86).

From tissue studies, deleterious mutations and inactivating structural rearrangements are present in 3-5% of metastatic prostate cancer (12, 87). Importantly, structural rearrangements in *MSH2* and *MSH6* are often large and complex, with breakpoints frequently in intronic and gene flanking regions (88). These characteristics present challenges for detection in ctDNA and tumor tissue alike. Assays that prioritize sequencing coverage of non-coding regions surrounding MMR genes are therefore helpful to identify these clinically relevant alterations that may sensitize to immunotherapy (89). Somatic hypermutation in metastatic prostate cancer is often associated with presence of these MMR gene defects (87, 89). In a recent non-randomized study assessing tumor mutational burden (TMB) *via* tissue biopsy, patients with high TMB mCRPC (defined as the FDA-approved cutoff ≥10 mutations per Mb) were shown to benefit greater from treatment with immune checkpoint inhibitors versus taxane chemotherapy (90). While TMB can be estimated in plasma cfDNA specimens (91, 92), thresholds for high TMB established in tissue studies may not be relevant for predicting checkpoint inhibitor sensitivity. Care should also be taken when interpreting any individual potentially clinically actionable mutation in patients with somatic hypermutation. Many represent subclonal passenger alterations (including in HRR-related genes), which are unlikely to result in overall disease sensitivity to targeted therapies.

#### ctDNA for clinical trial eligibility

Most contemporary basket and umbrella trials screen primary or metastatic tissue, typically formalin-fixed paraffin embedded (FFPE) specimens. However, plasma ctDNA screening is increasingly used to complement or replace existing screening procedures. Recent PARP inhibitor studies in mCRPC have endorsed ctDNA analysis alongside tumor tissue testing to cross-validate presence of HRR alterations (20–23). PC-BETS (IND.234) and ProBio are two ongoing biomarker-directed prospective mCRPC clinical trials utilizing cfDNA testing for treatment allocation (25, 93). Moving forward, efforts to improve reliability of alterations detected in ctDNA and the clinical contexts where ctDNA testing is appropriate will be essential for testing a broader range of hypotheses. Future biomarker-driven trials should also be aware of the bias cfDNA screening may introduce to patient selection. Trial eligibility predicated on minimum ctDNA% thresholds, or presence/absence of ctDNA can alter the risk profile of the study cohort, thereby impacting interpretation of targeted therapy efficacy.

### Acquired resistance and clonal evolution

Most patients with metastatic castration-naive prostate cancer exhibit exquisite sensitivity to ADT, with marked reductions in PSA and palliation of cancer-related symptoms. Despite this, continuous ADT results in inevitable treatment resistance and progression to mCRPC. In 75-80% of patients, sustained AR signaling drives ongoing tumor growth in mCRPC. Greater understanding of the genomic and non-genomic mechanisms of this AR-dependent phenotype have led to the development and successful implementation of next-generation ARPIs (94). In the remaining patients, AR-independent resistance mechanisms predominate. This subtype is characterized by aggressive disease features, low/absent AR protein expression or indifference to AR signaling. Importantly, prostate cancer exists on a spectrum between AR-dependent and AR-independent disease, and both can simultaneously exist within the same patient (60).

Clinically, this heterogeneity in intrinsic disease subtypes is evident in serial on-treatment imaging studies. Rarely are patterns of radiographic response uniform at all disease sites. On a single scan, certain lesions may grow despite treatment, while others shrink. This variation in response is indicative of distinct tumor populations that have developed acquired resistance to treatment. The ability of ctDNA to sample multiple cancer populations from spatially distinct metastatic foci presents opportunities to detect evolving genomic and epigenomic determinants of acquired treatment resistance (19). Specifically, the possibility of differentiating between AR-dependent and AR-independent metastatic prostate cancer is likely to have profound implications on optimal patient management.

#### AR resistance alterations

The *AR* gene is the most frequently altered locus in mCRPC (12–15). Genomic alterations include copy number amplification, ligand binding domain missense mutations and intronic structural rearrangements. These alterations are readily identifiable in ctDNA, and their presence is linked to clinical outcomes (95). *AR* gene body amplification in ctDNA is associated with shorter response to ARPI in patients with mCRPC, independent of prior treatment exposure (5, 41, 96–101). The magnitude of *AR* amplification also appears to be relevant, with high-level amplification associated with worse outcomes (5, 99). Notably, with repeated exposure to intensive AR-directed therapies, resistance mechanisms continue to converge upon the *AR* gene body and upstream enhancer, selecting for tumor populations with a greater number of *AR* copies (19, 102). Mutations in the *AR* are concentrated in hotspot regions within the ligand binding domain. The resultant conformational change induced by each specific mutation disrupts ligand specificity, resulting in inappropriate activation by older first-generation antiandrogens and endogenous steroids alike. including estrogen, progesterone, and glucocorticoids (103, 104). Data on whether these mutations confer resistance to newer ARPIs is conflicting (5, 37, 43, 96, 102). *AR* gene structural rearrangements can encode abnormal AR isoforms that lack the ligand binding domain, but still retain constitutive activity *via* intact N-terminal and DNA-binding domains. Patients exhibiting gene structural rearrangements predicted to truncate the ligand binding domain have been shown to portend shorter progression-free survival in mCRPC patients treated with ARPIs (5, 72, 102). Collectively, *AR* genomic alterations frequently co-occur, and cumulative burden in ctDNA is likely to have important clinical ramifications (43, 72). It is anticipated that with greater use of ARPI in the earlier mCSPC disease state, *AR* alterations will be more strongly selected for at mCRPC development. Convenient detection of these alterations in later disease states will be critical to interpreting the efficacy of emerging therapies targeting the AR-signaling axis, including AR degraders, N-terminal/DNA-binding domain inhibitors and spliceosome-targeted therapeutic agents (105).

#### Neuroendocrine differentiation

AR-independent prostate cancer is characterized by highly aggressive clinical features, including liver metastases, bulky lymphadenopathy, and lytic bone metastases. In most instances, it arises after long-standing intensive androgen suppression *via* ADT and/or ARPIs. Typically, these tumors begin as AR-dependent adenocarcinoma, before acquiring molecular events that promote tissue de-differentiation into small cell/neuroendocrine histologic features (*e.g.*, chromogranin and/or synaptophysin expression). The process of transitioning from one developmental pathway to another is known as lineage plasticity. While the circumstances that drive lineage plasticity in prostate cancer are incompletely understood, the central facet involves loss of tumor suppressors *RB1* and *TP53*, with accompanying transcriptional and epigenetic reprogramming (60).

Diagnosis of small cell/neuroendocrine prostate cancer (NEPC) usually requires tissue confirmation. More recently however, the role of ctDNA in detecting the emergence of this aggressive phenotype is being realized. Since biallelic loss of tumor suppressor genes in ctDNA is not specific to AR-independent prostate cancer (106), recent efforts have focused on using epigenomic techniques to improve NEPC detection. Adopting whole-genome bisulfite sequencing analysis from time-matched plasma cfDNA and metastatic tumor biopsies in a cohort of prostate adenocarcinoma and NEPC, Beltran et al. showed high concordance of ctDNA and tissue methylation patterns in patients with NEPC (107). The process of whole genome bisulfite sequencing is limited however by the requirement for high DNA input amounts. Newer approaches such as methylated DNA immunoprecipitation followed by high-throughput sequencing represent a highly sensitive method of obtaining genome-wide cfDNA methylation profiles, and have been successfully applied to identify NEPC with high specificity (108). Finally, cfDNA fragmentation patterns offer yet another alternate mechanism for NEPC diagnosis. In this method, nucleosome density at specific chromatin sites (*e.g.*, transcriptional start sites, transcription factor binding sites) can be inferred by analyzing the genomic location of cfDNA fragment end points. How consistently nucleosomes are positioned in these regulatory sites appears to be a surrogate measure of relative RNA abundance. This approach has been applied to quantify relative RNA abundance in key NEPC regulatory genes (109) and AR binding sites to determine AR activity in prostate cancer cells (19). Overall, these results hold great prospects for the use of cfDNA epigenomic patterns to track emergence of distinct prostate cancer subtypes associated with poor outcomes, permitting earlier implementation of proven platinum-based chemotherapy treatments and novel agents currently in clinical trials.

## Conclusion

**Figure 2:**
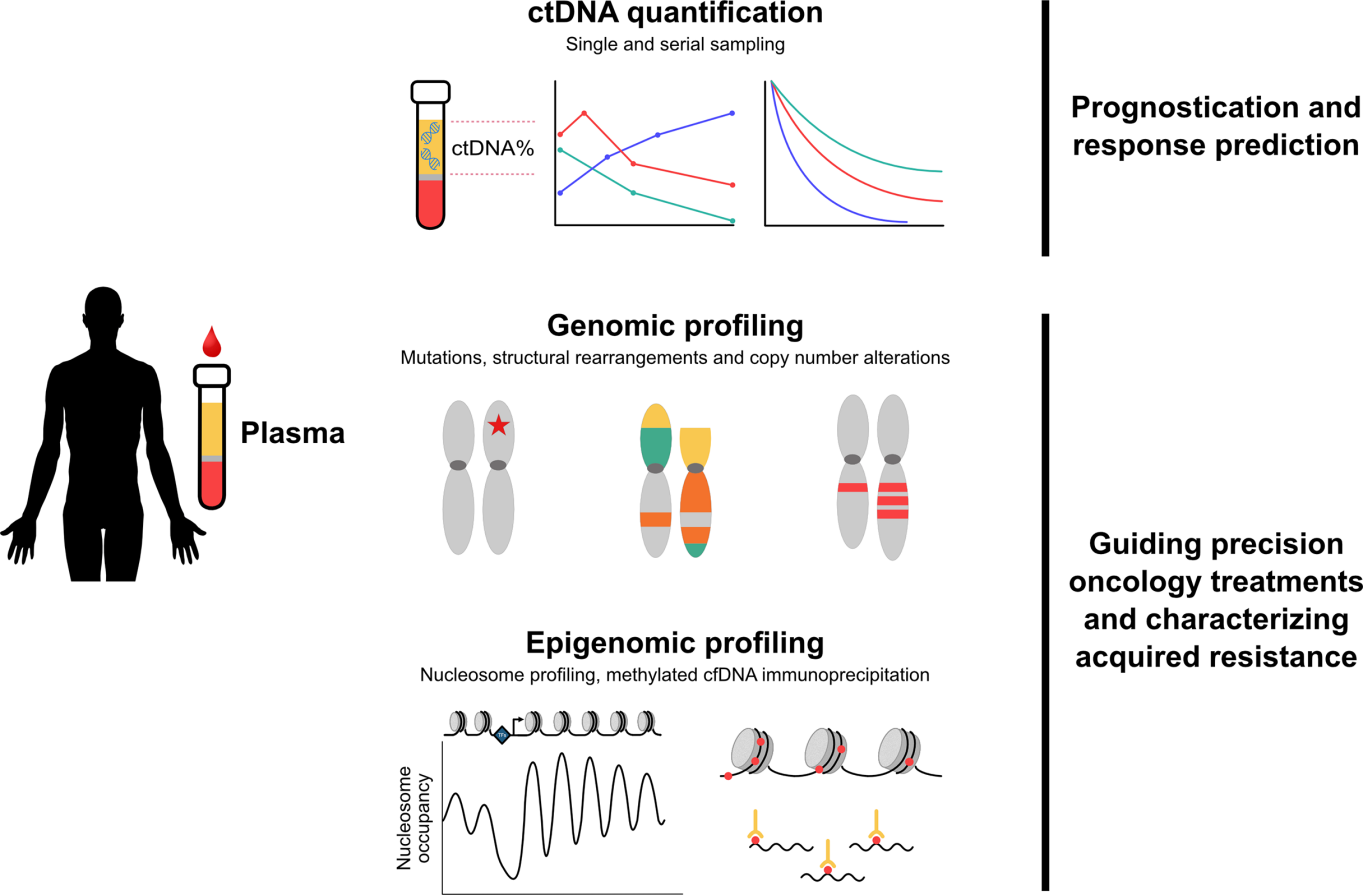
Overview of clinical applications of plasma ctDNA in metastatic prostate cancer. Quantification and genomic/epigenomic profiling of plasma ctDNA has identified multiple clinical applications relevant to metastatic prostate cancer. Top - ctDNA% is highly prognostic and outperforms standard clinical prognostic factors. Tracking changes in ctDNA% over time may represent a reliable early readout of treatment efficacy. Middle - characterization of mutations, structural rearrangements and copy number alterations guides use of targeted therapies and enables detection of evolving treatment resistance. Bottom - novel epigenomics approaches are capable of identifying aggressive subtypes of prostate cancer, including emergence of small cell/neuroendocrine disease. Abbreviations: cfDNA, cell-free DNA; ctDNA, circulating tumor DNA.

Plasma ctDNA analysis has emerged as a key application of liquid biopsy for metastatic prostate cancer, with a wide range of potential and proven clinical utility to inform on optimal patient management (**Figure 2**). ctDNA% is highly prognostic and outperforms standard clinical prognostic factors. Furthermore, the ability to track changes in ctDNA% over time may represent a reliable early readout of treatment efficacy. With commercial assays increasingly reporting ctDNA%, understanding the implications of both static and dynamic readouts of ctDNA% will enable future production of point-of-care assessment tools in the clinic. More broadly, ctDNA genotyping to guide use of targeted precision oncology therapies is now a reality. As the cost of sequencing depreciates and the overhead of informatic analysis improves, it will become increasingly important to use serial sampling methods to understand evolving mechanisms of acquired treatment resistance.

## Author contributions

This manuscript was drafted and critically reviewed by EMK, AWW, and KNC. All authors contributed to the article and approved the submitted version.

## Funding

This work was supported by a Prostate Cancer Foundation (PCF) Young Investigator Award and Killam Postdoctoral Fellowship to EMK.

## Conflict of interest

EMK reports consulting roles with Astellas Pharma, Janssen and Ipsen; honoraria from Astellas Pharma, Janssen, Ipsen and Research Review, and research funding from AstraZeneca (institutional), Merck (institutional) and Astellas Pharma (institutional). AWW reports commercial research grants from Janssen and ESSA Pharma, and honoraria from AstraZeneca, Astellas, Janssen, and Merck. KNC reports grants and personal fees from Astellas, AstraZeneca, Janssen, Merck, Novartis, Pfizer, Point Biopharma, Roche, and Sanofi.

The handling editor AA declared a past co-authorship with the author KNC.

## Publisher’s note

All claims expressed in this article are solely those of the authors and do not necessarily represent those of their affiliated organizations, or those of the publisher, the editors and the reviewers. Any product that may be evaluated in this article, or claim that may be made by its manufacturer, is not guaranteed or endorsed by the publisher.
